# Congenital Absence of the Nose

**Published:** 1856-07

**Authors:** 


Congenital Absence of the Nose.—New Rhinoplastic Operation.—M. Maisonneuve, of Paris. has lately operated upon a child which presented a singular deformity of the face at birth, viz., complete absence of the nasal prominence. This skilful surgeon devised and carried into successful execution, a new rhinoplastic operation, the ingenuity and simplicity of which are worthy the attention of surgeons.

Eugenie Marotte, seven months old, was born strong and well-formed, with the exception that she had no nasal prominence. In the place of this there was a plane surface, pierced by two small, round apertures, less than a line in diameter, and a little over an inch apart. This deformity not only rendered the child's face exceedingly grotesque, but also seriously embarassed the respiration, and, by consequence, the act of sucking.

It being very desirable to remedy these two difficulties, the parents brought the child to Paris with that intention.

There being no case on record, identical with this, the usual rhinoplastic procedures were unavailing. M. Maisonneuve planned the following ingenious operation.

On the 18th of May, 1855, the child being previously placed under the influence of chloroform, the surgeon made a transverse incision, a centimetre in length from each nasal aperture, from without, inwards. Two other vertical incisions, starting from the internal extremity of each of the former, were carried towards the free border of the lower lip, near which they approached each other and united into the form of a V. A narrow flap was thus formed by the latter incisions, and which included the entire thickness of the lip. This flap was dissected up and raised horizontally, so as to form the lower portion of the septum (*sous cloison*) of the artificial nose.

There then remained a factitious hare-lip; and its freshly divided edges were united by means of the twisted suture. In order, however, to obtain union, it became necessary that the space comprised between the nasal apertures should be lessened.by the whole breadth of the detached flap above mentioned—and that, consequently, a projecting fold should be formed, at the expense of the intervening integument, and which, supported by the artificial septum above mentioned, might thus form, naturally, a perfectly regular nasal prominence. In order fully to understand the ingenious and simple mechanism of this operation, it will suffice to try it upon a piece of paper; it will at once be evident that the desired result may be obtained.

Complete cure was not obtained without some slight accidents. The child, irritated by pain, cried almost constantly, and kept in nearly continual motion for twenty-four hours. In consequence of this, the uppermost points of suture became partially detached; this, however, gave the operator an opportunity to perfect the union of the artificial hare-lip. His method of doing this was by dividing the *orbicularis oris* muscle on each side of the wound by subcutaneous incision; and thus laceration of the adhering edges, by the contraction of the muscular fibres, was prevented.

In this way, union went on uninterruptedly, notwithstanding the constant movements of the little patient; and when she was removed from Paris, the cure was complete. The nose was very regularly formed, and the nostrils, being largely open, allowed free respiration.—*Boston Med. and Surg. Journal, from Gazette des Hopitaux, December,* 1855.

